# Arsenic Trioxide and the MNK1 Inhibitor AUM001 Exert Synergistic Anti-Glioblastoma Effects by Modulating Key Translational, Cell Cycle, and Transmembrane Transport Pathways

**DOI:** 10.3390/brainsci16020121

**Published:** 2026-01-23

**Authors:** Yue Hao, Charles Shaffer, Nanyun Tang, Valerie DeLuca, Angela Baker, Michael E. Berens

**Affiliations:** 1 Clinical Genomics and Therapeutics Division, Translational Genomics Research Institute, Phoenix, AZ 85004, USA; 2 Beckman Research Institute, City of Hope, Duarte, CA 91010, USA

**Keywords:** glioblastoma, arsenic trioxide, MNK1 inhibitor, synergy, glioblastoma stem cells

## Abstract

Background: The profound heterogeneity of glioblastoma and the often-limited efficacy of conventional treatments, including arsenic trioxide (ATO), underscore the urgent and critical demand for innovative combination strategies specifically designed to overcome treatment resistance. Methods: We evaluated the therapeutic effects of ATO as a single agent and in combination with the MNK1 inhibitor AUM001 across patient-derived xenograft (PDX) models and investigated molecular determinants of sensitivity and synergy. Our results demonstrated that GBM models resistant to ATO, particularly those of the mesenchymal subtype, are more likely to show synergistic cytotoxicity when AUM001 is added. The combination significantly reduces the frequency of glioblastoma stem cells (GSCs) compared to either drug alone, especially in ATO-resistant models. Results: These observations suggest that targeting the MNK1 pathway in conjunction with ATO is a promising strategy to specifically eradicate GSCs, which are major drivers of GBM recurrence and therapeutic failure. Transcriptomic analyses revealed that ATO sensitivity correlated with activated translation-related pathways and cell cycle processes, while synergistic responses to the combination were driven by distinct molecular signatures in different GBM subtypes. Overall, synergistic response to the combination therapy is more associated with cellular organization, amino acid transmembrane transporter activity, ion channels, extracellular matrix organization and collagen formation. Conclusions: Our findings highlight that specific molecular pathways and their activities, including those involving translation, cell cycle and ion transport, appear to modulate the synergistic efficacy of the ATO and AUM001 combination, thereby offering potential biomarkers for improved patient stratification in future GBM clinical trials of such ATO-based treatments.

## 1. Introduction

Glioblastoma (GBM) stands as the most aggressive and prevalent malignant primary brain tumor, presenting an exceptionally grim prognosis with a five-year survival rate of merely 6.8% [1]. Its formidable nature stems from profound heterogeneity, highly infiltrative growth patterns, and an intrinsic resistance to conventional treatment modalities, further compounded by the protective yet restrictive blood–brain barrier [2]. The persistent lack of newly approved agents for GBM underscores an urgent and critical demand for innovative therapeutic strategies capable of circumventing or overcoming the complex intrinsic and acquired resistance mechanisms that invariably lead to treatment failure and tumor recurrence [3]. The inherent heterogeneity of GBM is a primary driver of therapeutic failure. This includes the presence of distinct glioma stem cell (GSC) populations, which are recognized as pivotal contributors to tumor initiation, progression, and resistance to therapy [4,5,6,7]. These GSCs possess self-renewal capabilities and are particularly adept at evading standard treatments. Given this intricate molecular landscape and the adaptive resistance mechanisms characteristic of GBM, single-agent therapies often prove insufficient to achieve durable responses. Consequently, combination approaches that target multiple pathways or are specifically designed to overcome identified resistance mechanisms are considered essential for improving patient outcomes [8].

Arsenic trioxide (ATO), an FDA-approved drug, has a well-established role as a standard of care for myelodysplasia and relapsed or refractory acute promyelocytic leukemia (APL), where its efficacy is largely attributed to its ability to induce differentiation of leukemic cells [9]. While its precise mechanism of action in solid tumors is often considered idiopathic, ATO is recognized as a potent inducer of apoptosis in various malignant cells both in vitro and in vivo [10,11]. ATO induces profound cellular stress through a complex interplay of mechanisms, primarily initiating oxidative stress via the generation of reactive oxygen species (ROS) and depletion of antioxidants like glutathione [12]. This oxidative assault contributes to mitochondrial dysfunction, characterized by the loss of mitochondrial membrane potential, mitochondrial aggregation, and the release of pro-apoptotic factors such as cytochrome c [13]. Concurrently, ATO triggers endoplasmic reticulum (ER) stress, activating the unfolded protein response and disrupting calcium homeostasis, with ATF4 playing a key role in mediating crosstalk between the ER and mitochondria [14]. Furthermore, ATO causes DNA damage, both directly through ROS and indirectly by inhibiting DNA repair pathways, notably by targeting zinc finger proteins and downregulating key repair enzymes like BRCA1 and BLM [15]. These diverse stress signals converge to activate apoptotic pathways, involving caspases, Bcl-2 family proteins, and p53, ultimately leading to programmed cell death, which underpins ATO’s therapeutic efficacy, particularly in cancer treatment [16].

In the context of glioblastoma, ATO has demonstrated preclinical efficacy against GSCs, mediated through mechanisms such as the activation of apoptosis and autophagy, and the inhibition of the sonic hedgehog signaling pathway [17]. A critical aspect of ATO’s activity in GBM is the differential sensitivity observed across distinct GBM subtypes. Patient-derived GBM cells exhibit varied responses to ATO, with Mesenchymal (MES) GSCs show relative resistance, while non-MES GSCs demonstrating notable sensitivity [4]. This intrinsic differential response is a pivotal factor influencing ATO’s therapeutic potential in GBM. As a single agent, ATO’s efficacy in glioma is limited, necessitating alternative treatment strategies and clinical trial designs [18,19,20], such as combining ATO and temozolomide with radiation therapy to overcome resistance [21,22,23,24]. Understanding molecular signatures of vulnerability to ATO and its combinations would enable GBM clinical trial development. Analysis of patient data from such trials has revealed that proneural GBM responded more favorably to ATO, evidenced by longer overall and progression-free survival compared to other GBM subtypes [4]. There’s growing recognition of the need for genetic-based patient selection criteria to improve clinical trial outcomes [25].

Given the limitations of ATO as a monotherapy and the identified role of mRNA translation in mediating differential responses to it, targeting pathways that regulate protein synthesis presents a rational strategy to enhance ATO’s efficacy in GBM. Mitogen-activated protein kinase (MAPK)-interacting kinases (MNKs), specifically MNK1 and MNK2, function as crucial downstream effectors of the MAPK pathway [26]. Their primary role involves the phosphorylation of the eukaryotic initiation factor 4E (eIF4E) at Ser-209, a pivotal step in cap-dependent mRNA translation initiation [26]. This phosphorylation event critically regulates the synthesis of numerous proteins involved in cell growth, survival, and proliferation [27,28,29]. Dysregulation of MNK activity and subsequent aberrant upregulation of eIF4E phosphorylation are frequently observed across various cancer types, leading to increased synthesis of oncogenic and anti-apoptotic proteins, thereby promoting uncontrolled cell proliferation, enhanced cell survival, and the development of therapeutic resistance [30]. In GBM, the inherent resistance of Mesenchymal (MES) GSCs to ATO has been directly linked to the overexpression of genes associated with translation initiation, general translation, and the upregulation of eIF4E within these specific GSC populations. Critically, the differential response of GSCs to arsenic trioxide therapy has been demonstrated to be intricately regulated by MNK1 and mRNA translation [4]. Treatment with ATO in combination with MNK1 inhibitor, not only sensitizes models initially resistant to ATO but displays a preferential effect on the GSC population [4,31]. AUM001 (previously ETC-206) is an active inhibitor of MNK1 [32] which is currently in clinical trial in combination with pembrolizumab or irinotecan in patients with colorectal cancer (NCT05462236), with its pharmacokinetics demonstrate ideal pharmaceutical properties [33]. A novel therapy utilizing AUM001 in combination with ATO may serve to further sensitize malignant glioma through inhibition of the MNK1-eIF4E axis.

In this study, we comprehensively evaluated the therapeutic effects of ATO. We tested its efficacy as a single agent as well as in combination with the MNK1 inhibitor, AUM001, across a panel of 14 GBM patient-derived xenograft (PDX) models [34]. Beyond assessing efficacy, our research delved into the underlying mechanisms by identifying key molecular pathways associated with sensitivity versus resistance to ATO monotherapy. Furthermore, we aimed to elucidate the molecular determinants that contribute to synergistic, as opposed to merely additive, cytotoxic effects, particularly for the ATO and AUM001 combination. Alongside this main combination, we also investigated the potential for synergistic interactions between ATO and nutraceuticals Chrysin or Silibinin [35,36], which are naturally occurring flavonoids and flavonolignans of reported anti-glioma activity. Based on these findings, we propose potential biomarkers to refine patient selection and stratification strategies for future clinical trials of ATO-based combination therapies in GBM.

## 2. Materials and Methods

### 2.1. GBM PDX Models and Short-Term Culture

Glioblastoma patient-derived xenografts (PDX) were acquired from the Mayo Clinic [29]. These models were cultured as spheroids in DMEM/F-12 (1:1), Glutamax^TM^ (Gibco, 10565018, Thermo Fisher Scientific, Waltham, MA, USA), supplemented with 2% (*v*/*v*) B27 (Gibco, 17504044), 1% (*v*/*v*) N2 (Gibco, 17502048), 20 ng/mL EGF (Peprotech, AF-100-15, Thermo Fisher Scientific, Waltham, MA, USA), 20 ng/mL FGF (Peprotech, AF-100-18B) and 2 µg/mL Gentamicin (Gibco, 15750060). Cells were dissociated into a single cell suspension with Accutase for seeding (Innovative Cell Technologies, AT-108, San Diego, CA, USA).

### 2.2. Single Agent and Synergy Treatment

**Figure 1:**
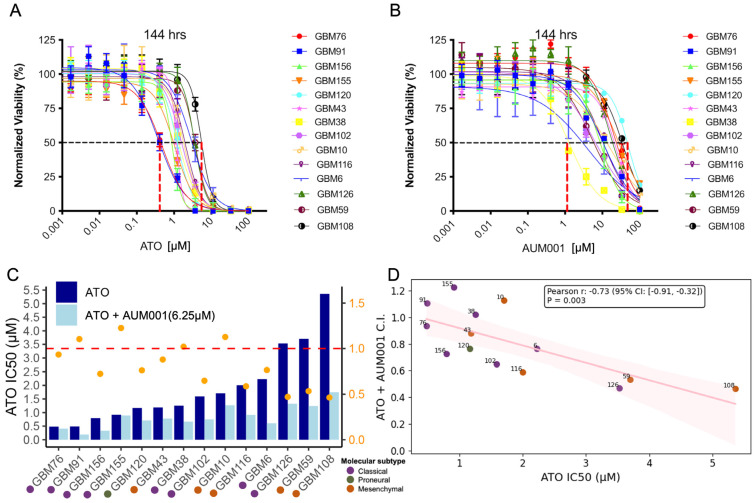
Cytotoxic effects of ATO and AUM001 as single agents and in combination in GBM PDX cultures. Dose–response curves of various GBM models treated with increasing concentrations of ATO (**A**) or AUM001 (**B**) for 144 h. Cell viability is expressed as a percentage of untreated (control) cells. Data points represent the mean ± SD from representative experiments. The two red dashed lines marked the range between the lowest and the highest IC50. (**C**) Bar chart displaying the ATO IC50 values (µM) for a panel of GBM PDX cultures when treated with ATO alone (dark blue bars) or ATO in combination with 6.25 µM AUM001 (light blue bars). Orange dots represent the C.I. values for the ATO and AUM001 (6.25 µM) combination, plotted on the secondary y-axis. The red dashed line indicates a C.I. value of 1. Dots below the dashed line (C.I. < 1) represent synergistic response. The molecular subtypes of the GBM are labeled. (**D**) Scatter plot illustrating the correlation between ATO IC50 (µM) values and the C.I. for ATO + AUM001 (6.25 µM) across the tested GBM PDX cultures. The solid red line represents the linear regression, and the shaded pink area indicates the 95% confidence interval. The Pearson correlation coefficient (r) and *p*-value are displayed. Individual GBM PDX culture identifiers are shown next to their respective data points.

Cells were seeded at 1000 cells/well in 384-well plates in culture media lacking Gentamicin and allowed to adhere overnight. For single agent treatments, cells were treated with ATO or AUM001 at 1:3 serial dilutions ranging from 100 µM–0.56 nM. For combination treatments, cells were treated with ATO ranging from 100 µM–0.56 nM in addition with AUM001 (25, 12.5, 6.25, or 3.125 µM), Chrysin (10 µM) or Silibinin (12.5 or 25 µM). Following a treatment duration of 72- or 144-h, cell viability was measured with CellTiter-Glo^®^ Luminescent Cell Viability Assay (Promega, G7570, Madison, WI, USA) on Envision 2105 Multi Mode Plate Reader (Perkin Elmer, Waltham, MA, USA). The concentration ranges for ATO and AUM001 were determined based on their respective IC50 values across the panel of GBM PDX cell lines to ensure a comprehensive evaluation of both sensitive and resistant lines in vitro. Combination Index (C.I.) that quantifies the synergistic effects was calculated using the Chou–Talalay method [37], where a C.I. < 1 indicates synergy, C.I. around 1 indicates an additive effect, and C.I. > 1 indicates antagonism. For ATO + AUM001, out of the total 12 × 4 = 48 drug combination concentrations tested, the lowest C.I. was shown in Figure 1 and was listed in Table S1.

### 2.3. Extreme Limiting Dilution Analysis

**Figure 2:**
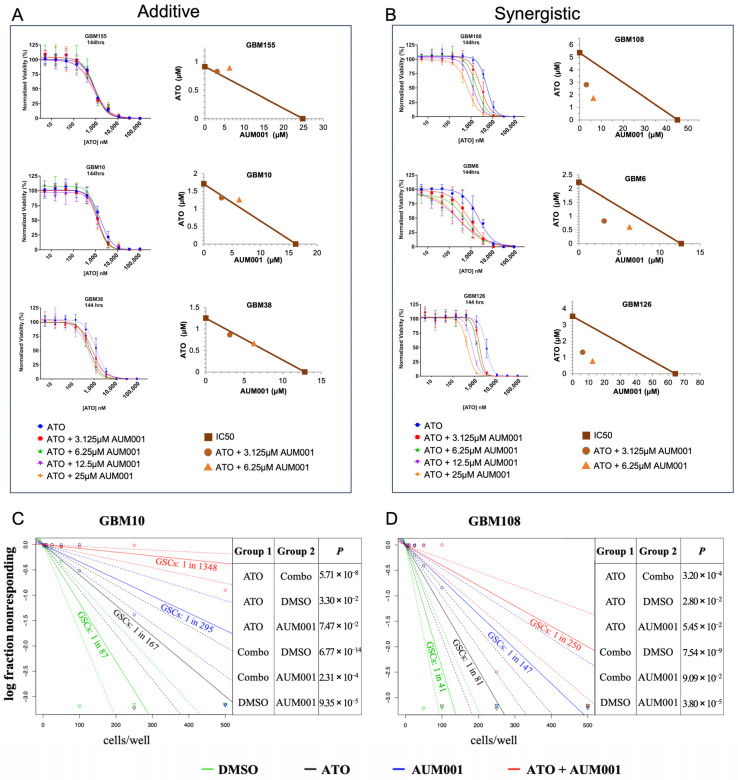
Characterization of ATO and AUM001 combination effects in additive and synergistic GBM PDX models and impact on GSC frequency. (**A**) Dose–response curves of GBM PDX models GBM155, GBM10, and GBM38, classified as exhibiting additive interactions. Cells were treated with increasing concentrations of ATO alone or in combination with fixed concentrations of AUM001 (3.125 µM, 6.25 µM, 12.5 µM, 25 µM) for 144 h. Cell viability is expressed as a percentage of untreated control cells. Isobolograms show C.I. values for ATO in combination with varying AUM001 concentrations. (**B**) Dose–response curves of GBM PDX models GBM108, GBM6, and GBM126, classified as exhibiting synergistic interactions. Cells were treated as described in (**A**). Isobolograms show C.I. values. (**C**) ELDA of GBM10 cells treated with DMSO (vehicle control), ATO, AUM001, or the combination of ATO and AUM001. The graph plots the log fraction of stem cell-positive wells (non-responding) against the number of cells seeded per well. Calculated GSC frequencies for each treatment group are indicated. The table shows *p*-values for comparisons between different treatment groups. (**D**) ELDA of GBM108 cells treated as described in (**C**). Calculated GSC frequencies and *p*-values for comparisons between treatment groups are shown. Data points in A and B represent the mean ± SD from representative experiments. Lines in C and D represent the fit of the ELDA model.

Cells were seeded into 96-well plates at 1, 5, 10, 25, 50, 100, 250, and 500 cells/well using a SH800S cell sorter (Sony, Tokyo, Japan). The concentrations for the ELDA were established based on the average IC50 of ATO (approximately 1 µM) across all PDX models and the observation that 6.25 µM of AUM001 consistently yielded the most robust synergistic response (Figure 2). Consequently, this combination was selected to evaluate the impact of dual inhibition on the self-renewal capacity of the PDX cell lines. Cells were then incubated overnight at 37 °C and then treated with either DMSO control, 1 µM ATO, 6.25 µM AUM001, or combination. After 7 days, wells were scored for formation of neurospheres using Cytation5 Cell Imaging Multimode Reader (Biotek, Winooski, VT, USA), with spheres greater than 150 nm in diameter marking positive formation. Extreme limiting dilution analysis was performed using software available https://bioinf.wehi.edu.au/software/elda/ (URL accessed on 22 January 2026) as previously described [38].

### 2.4. Western Blot

Cells were lysed in RIPA buffer containing protease and phosphatase inhibitors. Protein concentrations were determined using the Pierce BCA Protein Assay Kit (Thermo Scientific, 23225, Waltham, MA, USA). 45 µg of protein was size separated by 4–12% Bis-Tris NuPAGE gel (Invitrogen, NP0336BOX, Waltham, MA, USA). Separated proteins were transferred to a polyvinylidene (PVDF) membrane using Invitrogen iBlot 2 Gel Transfer Device (Invitrogen, IB21001), blocked in 5% BSA for 1 h at room temperature, then incubated with primary antibodies at 4 °C overnight. The antibodies against MNK1 (#2195) and B-Actin (#4970) were purchased from Cell Signaling Technology (Danvers, MA, USA). Specific signal was detected with species-appropriate HRP-conjugated secondary antibody (Promega) using Super Signal West Dura Extended Duration Substrate (Thermo Scientific, 34075) and imaged using a LI-COR Odyssey Fc imaging system (LI-COR, Lincoln, NE, USA). Densitometry and antibody information are displayed in Supplemental Table S2.

### 2.5. Differential Expression and GSEA Analyses Using mRNA Expression Data of Patient-Derived and Primary Glioblastoma Samples

Figure S2 shows the bioinformatics and statistical analysis workflow. GBM PDX model mRNA expression data was downloaded from CBioPortal [34]. Differential expression analysis for synergistic and additive PDX models was conducted using DESeq2 v1.42.1 [39] in R 4.3.3. GSEA analyses were performed using the R package clusterProfiler v4.10.1 [40,41]. For GSEA of PDX models, genes were ranked using the Spearman correlation coefficient between baseline gene expression and either ATO sensitivity IC50 or the Combination Index (C.I.). Enrichment was determined using the Reactome and GO Molecular Function gene sets from MSigDB [42]. Pathways were considered significantly enriched if the False Discovery Rate (FDR)-adjusted *p*-value was <0.05. Statistical overrepresentation tests for enriched Gene Ontology terms were done using PANTHER database [43]. For the PANTHER overrepresentation tests, gene significance was determined by an ANCOVA model (F-statistics *p* ≤ 0.1). Overrepresentation was assessed using Fisher’s Exact test with FDR correction, reporting terms with a fold enrichment > 1.

ATO clinical trial gene expression data is from previous publications [4,19]. In this dataset, 16 patients are ATO resistant, and 4 patients are ATO sensitive. Transcript per million (TPM) expression value matrix of 20 patients were used for single-sample GSEA analyses, which were performed using the publicly available implementation of ssGSEA method on Rpubs by Pranali S [44].

### 2.6. Statistical Analysis and Graphical Representation

All experiments were conducted with at least three independent experiments, and each biological run was repeated four times (3 biological replicates × 4 technical repeats). Error bars indicate mean ± standard deviation (SD). Graphs were generated using GraphPad Prism 10 software (Version 10.2.3, GraphPad Software LLC, Boston, MA, USA) and in R version (4.3.3) using the ggplot2 package version 3.5.1 [45].

## 3. Results

### 3.1. ATO Resistance PDX Models Are More Likely to Have Synergistic Effects with AUM001

The cytotoxic effects of Arsenic Trioxide (ATO) and the MNK1 inhibitor AUM001, both individually and in combination, were evaluated across short-term cultures from a panel of glioblastoma (GBM) patient-derived xenografts (PDX). The duration of treatment was set at 144 h, as preliminary assays indicated that AUM001 requires extended exposure to elicit a measurable inhibitory effect. At 144 h post-treatment, both ATO (Figure 1A) and AUM001 (Figure 1B) demonstrated dose-dependent inhibition of cell viability. Notably, different cell lines exhibited varying sensitivities to each compound.

The half-maximal inhibitory concentration (IC50) for ATO was determined for each cell line and is presented in Figure 1C (see also Table S1 and Figure 2). The IC50 values for ATO varied 10-fold across the models, ranging from approximately 0.5 µM in GBM76 to over 5 µM in GBM108. The most sensitive PDX cell lines were all derived from the classical type of GBM tumor, while the most resistant cell lines are derived from the mesenchymal type of GBM [34], although exceptions existed. When ATO was combined with the MNK1 inhibitor AUM001 (6.25 µM), a general trend towards a reduction in the ATO IC50 was observed across most cell lines, suggesting a potential synergistic or additive effect of the combination treatment (Figure 1C). Here we show ATO IC50 with 6.25 µM of AUM001 as it represented the optimal concentration where the strongest synergistic effects with ATO were observed across most PDX cell lines (Figure 2). In cell lines such as GBM108, GBM6, and GBM126, the addition of AUM001 led to a noticeable decrease in the ATO IC50. The ATO and AUM001 combination effect was indicated by the Combination Index (C.I., see also Figure 2), which is more likely to drop below 1 in cell lines that are more resistant to single agent ATO. C.I. values < 1 or ~1 indicate synergism or additive effect, respectively. Thus, we observe higher synergistic effects in mesenchymal-like ATO resistant lines. A correlation analysis was performed to investigate the relationship between the sensitivity to ATO alone (ATO IC50) and the combination effect of ATO and AUM001 (Figure 1D). The analysis revealed a statistically significant negative correlation (Pearson r = −0.73, *p*-value = 3.13 × 10^−4^) between the ATO IC50 and the ATO + AUM001 C.I. value. This indicates that cell lines initially more resistant to ATO (higher ATO IC50) tended to exhibit a stronger synergistic interaction (lower C.I.) when treated with the combination of ATO and AUM001.

### 3.2. Combined ATO and AUM001 Treatment Elicits Additive to Synergistic Cytotoxicity and Effectively Targets Glioblastoma Stem Cells in PDX Models

Using 14 patient-derived xenograft (PDX) models that exhibit either predominantly additive or synergistic responses, we investigated the nature of the interaction between ATO and AUM001. Figure 2A displays the dose–response curves for GBM models GBM155, GBM10, and GBM38, which demonstrated primarily additive effects when ATO was combined with increasing concentrations of AUM001. In these models, the addition of AUM001 resulted in a leftward shift in the ATO dose–response curve, indicative of increased potency, but the overall interaction was classified as additive based on C.I. values. C.I. values for specific concentrations are shown as isobolograms. Conversely, Figure 2B illustrates the effects in GBM models GBM108, GBM6, and GBM126, where the combination of ATO and AUM001 produced synergistic cytotoxicity. In these synergistic models, the co-administration of AUM001 with ATO led to a more pronounced reduction in cell viability than would be expected from an additive effect, as evidenced by their respective C.I. values (isobolograms). For example, in the GBM108 model, the combination treatment markedly enhanced the cytotoxic effect of ATO across various AUM001 concentrations. Supplementary Figure S1 shows the dose response curves and isobolograms of the other PDX models that we tested.

To assess the impact of these treatments on the glioblastoma stem cell (GSC) population, Extreme Limiting Dilution Analysis (ELDA) was performed on GBM10 and GBM108 models. In the GBM10 model (Figure 2C), treatment with ATO alone or AUM001 alone resulted in a modest reduction in GSC frequency compared to the DMSO control (GSCs: 1 in 87 for DMSO, 1 in 167 for ATO, and 1 in 295 for AUM001). However, the combination of ATO and AUM001 led to a much more substantial decrease in GSC frequency, with an estimated 1 GSC in 1348 cells. A pronounced effect on GSC frequency was also observed in GBM108 model (Figure 2D). While ATO and AUM001 monotherapies reduced GSC frequency (GSCs: 1 in 41 for DMSO, 1 in 81 for ATO, and 1 in 147 for AUM001), the combination treatment decreased the GSC population to an estimated 1 GSC in 250 cells. The ELDA results showed a potent reduction in GSC frequency with the ATO + AUM001 combination, experimentally validating the therapeutic concept proposed by Bell et al. (2018) [4]. The strong combination effect observed against GSCs with ATO + AUM001 is likely due to AUM001 inhibiting MNK1, thereby blocking a key pathway that GSCs (especially resistant mesenchymal GSCs) use to evade ATO’s cytotoxic effects. By inhibiting MNK1, AUM001 sensitizes the GSCs to ATO, leading to a more profound depletion of this critical tumor-initiating and therapy-resistant cell population. These ELDA results suggest that targeting the MNK1 pathway in conjunction with ATO warrants further investigation as a potential strategy to address the GSC population, which has been implicated as a significant contributor to GBM recurrence and therapeutic resistance.

### 3.3. Clinical Data Validated That Translation-Related Pathways Are Enriched in ATO-Sensitive GBM Samples

**Figure 3:**
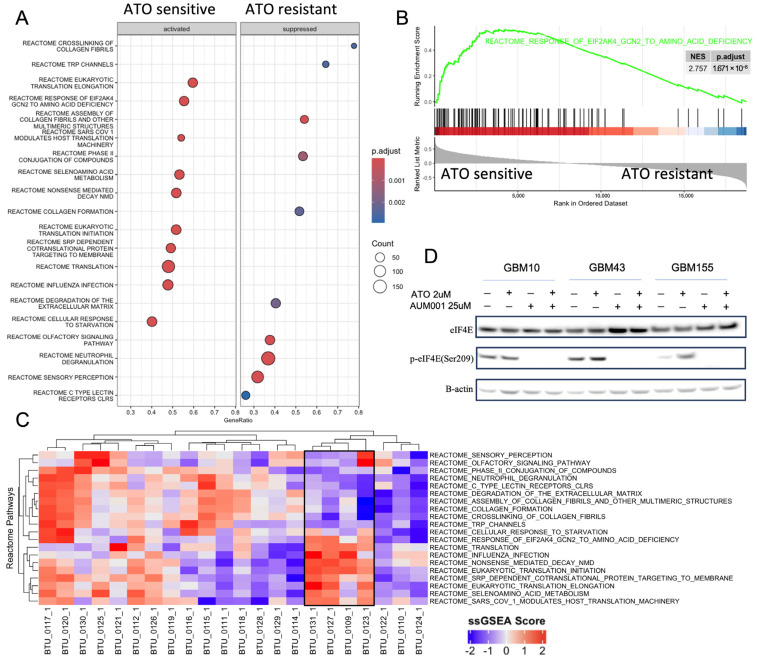
ATO sensitivity is associated with active translation pathways, and MNK1 inhibition reduces eIF4E phosphorylation in GBM cells. (**A**) GSEA dot plot of Reactome pathways comparing ATO-sensitive and ATO-resistant GBM PDX models. Dots represent pathways, with color intensity indicating adjusted *p*-value and size corresponding to the number of genes in the set. GeneRatio reflects the proportion of genes from the pathway found among the differentially expressed genes. Pathways are grouped by whether they are activated in sensitive lines or suppressed in sensitive lines (enriched in resistant lines). (**B**) GSEA enrichment plot for the “REACTOME RESPONSE OF EIF2AKA GCN2 TO AMINO ACID DEFICIENCY” pathway, showing significant enrichment in ATO-sensitive models (Normalized Enrichment Score = 2.757, p.adjust = 1.671 × 10^−8^). (**C**) Single-sample GSEA results for ATO clinical trial patient gene expression data using the top 20 Reactome pathways (NCT00275067). The four ATO sensitive samples are highlighted in a box. (**D**) Western blot analysis of total eIF4E and phosphorylated eIF4E (p-eIF4E(Ser209)) levels in GBM10, GBM43, and GBM155 cell lines. Cells were treated for 24 h with vehicle control (−, Arsenic Trioxide (ATO, 2 µM), AUM001 (25 µM), or the combination of ATO (2 µM) and AUM001 (25 µM). β-actin was used as a loading control. The results show that AUM001 treatment, alone or in combination with ATO, effectively inhibits eIF4E phosphorylation. In GBM43 and GBM155 cells, ATO treatment alone increased p-eIF4E levels, an effect abrogated by co-treatment with AUM001. Total eIF4E levels remained largely unchanged across conditions.

To elucidate candidate molecular mechanisms underlying differential sensitivity to ATO across the 14 PDX models, using transcriptomic data we calculated spearman correlations between genes and the ATO sensitivity (Figure S2), then performed Gene Set Enrichment Analysis (GSEA) using the ranked gene list by expression–sensitivity correlation Analysis using MSigDB [42]. Reactome pathways revealed distinct enrichment patterns in ATO sensitive vs. resistant lines (Figure 3A). Notably, pathways associated with mRNA translation, including “EUKARYOTIC TRANSLATION INITIATION”, “EUKARYOTIC TRANSLATION ELONGATION” (94 genes) and “REACTOME TRANSLATION” were significantly activated in ATO-sensitive relative to ATO-resistant models. Further supporting the role of translational regulation, the “RESPONSE OF EIF2AKA GCN2 TO AMINO ACID DEFICIENCY” pathway (102 genes) showed significant enrichment in ATO-sensitive models (NES = 2.757, p.adjust = 1.671 × 10^−8^) (Figure 3B). EIF2A (also known as GCN2) and eIF4E are both crucial proteins involved in translation regulation, but they function through distinct mechanisms and pathways, diverging primarily at the stage of translation initiation. The fact that different components of the translation initiation machinery emerged as enriched suggests that the synergistic effect of ATO and AUM001 could be due to efficacy changes in translation initiation during protein synthesis. Pathways related to collagen formation and extracellular matrix (ECM) organization appeared to be suppressed in ATO-sensitive lines and upregulated in the ATO-resistant phenotype (Figure 3A). The top 20 significant Reactome pathways in Figure 3C successfully cluster the four ATO-sensitive patient samples [4,22] that form a cluster in the single-sample GSEA results shown in Figure 3C. These four samples show similar expression patterns: highly expressed in translation-related pathways and downregulated in collagen- and ECM-related pathways. While these patterns align with our preclinical PDX data, the limited patient numbers mean this clinical correlation is currently underpowered and serves primarily to provide a preliminary clinical context for our mechanistic observations. It is worth noting that in the 16 ATO-resistant patient samples, the expression patterns vary widely, depicting the complex mechanisms behind ATO as a single agent.

### 3.4. eIF4E Phosphorylation Was Completely Inhibited but Total eIF4E Levels Were Not Impacted with Treatment of AUM001 Within GBM Cells

Given the established link between MNK1, eIF4E phosphorylation, and mRNA translation in ATO response, we investigated the effects of ATO and the MNK1 inhibitor AUM001, alone and in combination, on the phosphorylation of eIF4E at Serine 209 (p-eIF4E(Ser209)) in GBM10, GBM43, and GBM155 cell lines with varying ATO sensitivity (Figure 3D and Figure S4). Western blot analysis revealed that total eIF4E and β-actin levels remained constant across all treatments and cell lines (GBM10, GBM43, and GBM155), confirming that neither ATO nor AUM001 altered overall eIF4E expression. In contrast, eIF4E phosphorylation at Serine 209 (p-eIF4E) exhibited distinct, treatment-dependent shifts. While GBM10 cells showed stable p-eIF4E levels following ATO treatment, GBM43 and GBM155 cells responded with a noticeable upregulation of p-eIF4E. Despite these differences in ATO response, AUM001—both as a monotherapy and in combination—effectively abolished p-eIF4E across all three lines. Notably, AUM001 completely overrode the stimulatory effect of ATO in the combination groups, nearly abrogating the phosphorylation signal. In summary, these results demonstrate that MNK1 inhibition by AUM001 potently suppresses eIF4E activation, even when induced by ATO. The observed inhibition of eIF4E phosphorylation points toward the MNK1-eIF4E axis as a potential contributor to the varying sensitivity seen in ATO combination treatments.

### 3.5. Gene Expression Signatures Differentiate Synergy Responses Across GBM Subtypes

We performed differential gene expression analysis of the 14 PDX cell lines using a DESeq2 [39] multifactor design which accounts for both ATO sensitivity and ATO + AUM001 synergy to investigate the molecular mechanisms underlying the response to ATO and its combination with AUM001 (Figure S2). This analysis was conducted separately for non-mesenchymal and mesenchymal-like GBM PDX cell lines to identify subtype-specific signatures.

**Figure 4:**
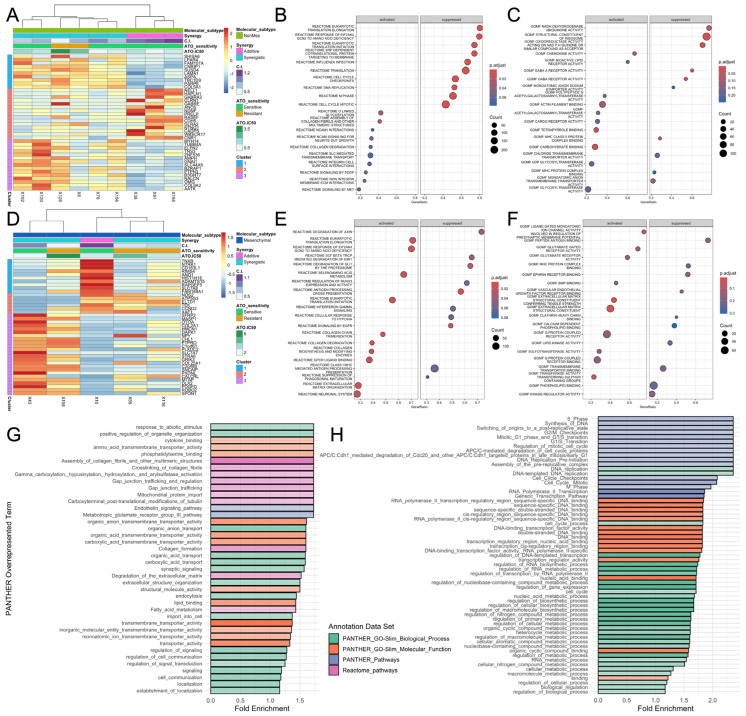
Transcriptomic Analysis Reveals Molecular Correlates of ATO and AUM001 Synergy in GBM PDX Models. (**A**–**F**) Differential gene expression and pathway enrichment analysis using a DESeq2 multifactor model incorporating both ATO sensitivity and ATO + AUM001 Synergy as factors. The top 40 differentially expressed (DE) genes are depicted in heatmaps where rows (genes) and columns (PDX models) are clustered based on expression similarity. DE genes from the Synergy contrast (Synergistic vs. Additive) were used to generate a ranked gene list for GSEA. (**A**–**C**) Analysis of PDX models derived from non-mesenchymal tumors. (**A**) Heatmap of the top 40 DE genes. Annotations indicate Molecular Subtype, ATO sensitivity, Synergy (C.I.), and resulting clusters. (**B**) Enriched Reactome pathways from GSEA. (**C**) Enriched Gene Ontology (GO) Molecular Functions from GSEA. (**D**–**F**) Analysis of PDX models derived from mesenchymal-like GBM molecular subtype. (**D**) Heatmap of the top 40 DE genes. (**E**) Enriched Reactome pathways from GSEA. (**F**) Enriched GO Molecular Functions from GSEA. In (**B**,**C**,**E**,**F**), the dot size corresponds to the number of genes in the set, and color intensity reflects the statistical significance (FDR). (**G**,**H**) PANTHER Overrepresentation Fisher’s exact tests identifying enriched Gene Ontology terms among genes significantly contributing to synergy. Gene significance was determined by an ANCOVA model with synergy C.I. as the response, gene expression TPM as variate, and molecular subtype and ATO sensitivity (IC50) as two covariates (F statistics *p* ≤ 0.1). Genes with the expression variable F statistics *p* ≤ 0.1 were used in the overrepresentation tests. (**G**) GO terms overrepresented among genes that are highly expressed in synergistic models (C.I. < 1) and significantly contribute to synergy (Spearman ρ ≤ −0.2). (**H**) GO terms overrepresented among genes that are highly expressed in additive models (C.I. ~1) and significantly contribute to this effect (Spearman ρ ≥ 0.2). Bar plots show fold enrichment. Annotation Data Sets used for PANTHER include PANTHER GO-Slim Biological Process, PANTHER GO-Slim Molecular Function, PANTHER Pathways, and Reactome pathways.

In the non-mesenchymal tumor-derived cultures, hierarchical clustering of the top 40 differentially expressed (DE) genes revealed distinct expression profiles that segregated them (Figure 4A). Notably, models exhibiting synergistic responses to the combination treatment (C.I. < 1) tended to cluster together, indicating a shared gene expression signature associated with synergy. A separate cluster was observed for models experiencing an additive response.

GSEA of Reactome Pathways (Figure 4B) and Gene Ontology (GO) Molecular Functions (Figure 4C) distinguishing between non-mesenchymal models (n = 9) showing Synergistic vs. Additive responses revealed that synergistic responses were associated with the activation of pathways related to cell signaling, surface interactions, immune response and binding activities, including “NCAM1 interactions”, “signaling by MET”, “signaling by PDGF”, “integrin cell surface interactions”, “chemokine activity”, “actin filament binding” and “carbohydrate binding”. Conversely, the most significantly suppressed pathways in synergistic lines were related to protein synthesis and cell cycle progression, such as “translation initiation”, “translation elongation”, “DNA replication” and “M phase”, as well as several transporter activities (“monoatomic anion transmembrane transporter activity”, “chloride transmembrane transporter activity”).

In mesenchymal GBM models (n = 5) GSEA results pointed towards a differentiating set of biological processes between Synergy and Additive responses. Enriched Reactome pathways in the activated set were dominated by pathways related to translation, the extracellular matrix and cell signaling, including “translation initiation”, “extracellular matrix organization” and “GPCR ligand binding” (Figure 4E). In contrast, the most significantly suppressed pathways were related to protein synthesis (“translation elongation”, “translation initiation”) and immune response (“antigen processing”, “interferon gamma signaling”). The GO Molecular Function analysis strongly supported these findings, with the most significant activated terms being “ion channel activity”, “extracellular matrix structural constituent” and “conferring tensile strength”, alongside various receptor activities like “G protein coupled receptor activity” and “glutamate receptor activity” (Figure 4F). The most significantly suppressed function was “peptide antigen binding”, consistent with the suppression of immune-related pathways. These results demonstrate that distinct transcriptomic profiles and biological pathway activities are associated with ATO sensitivity and its synergy with AUM001 in different tumor molecular contexts.

To further control for the confounding factor of ATO single agent sensitivity and to identify biological processes robustly contributing to the synergistic effect, we conducted PANTHER Overrepresentation Fisher’s exact tests. These tests utilized genes found to significantly contribute to synergy based on an analysis of covariance (ANCOVA) model with synergy C.I. as the response, gene expression TPM as variate, and molecular subtype and ATO sensitivity (IC50) as two covariates (Figures S2 and S3). Genes highly expressed in synergistic models (low C.I.) that significantly contributed to synergy (Spearman ρ ≤ −0.2, F statistics *p* ≤ 0.1) were found to be overrepresented in GO terms such as “positive regulation of organelle organization”, “amino acid transmembrane transporter activity”, and “collagen formation” (Figure 4G). Conversely, genes whose higher expression was associated with a lack of synergy (or an additive effect) and significantly contributed to this outcome (Spearman ρ ≥ 0.2, F statistics *p* ≤ 0.1) were overrepresented in GO terms including “switching of origins to a pre-replicative state”, “M/G1 phase transition of mitotic cell cycle”, and “DNA binding transcription factor activity” (Figure 4H). These analyses pinpoint distinct biological pathways and functions whose expression levels are critical determinants of the synergistic efficacy of the ATO and AUM001 combination.

Specifically, Figure 4G indicates that genes highly expressed in synergistic models are overrepresented in GO terms such as “amino acid transmembrane transporter activity”, “organic anion transmembrane transporter activity”, “organic acid transmembrane transporter activity”, “carboxylic acid transmembrane transporter activity”, “monovalent inorganic cation transmembrane transporter activity” and “ion transmembrane transporter activity” among those enriched in synergistic models, which agrees with the enriched Molecular Functions in Figure 4C,F. These terms are directly related to the function of ion channels and other transmembrane transporters that move ions and molecules across cellular membranes. Genes highly expressed in additive models are dominated by terms related to cell cycle progression and DNA replication. There is no indication that ion channel-related terms are significantly overrepresented in the additive models (Figure 4H). Therefore, our results suggest that increased expression of genes involved in ion transport and general transmembrane transporter activity is associated with a synergistic response to the ATO and AUM001 combination in these GBM models.

### 3.6. Chrysin and Silibinin Sensitize GBM Cell Lines to ATO and Improve ATO Therapeutic Potency

Naturally occurring flavonoids are themselves non-toxic and have demonstrated potentiating anti-glioblastoma activities of chemotherapeutic drugs [35]. We conducted combinatory assays to test for improved therapeutic potency of ATO (Figure S5). Across these three cell lines tested (GBM10, GBM43 and GBM155), Chrysin alone exhibited minimal cytotoxicity, while its combination with 1.24 µM ATO resulted in much lower cell viability compared to ATO monotherapy, with the most substantial enhancement observed in GBM43 cells (28% viability decrease). A separate set of experiments evaluated ATO in combination with Silibinin, using different concentrations of both agents for each cell line (Figure S5). Similar to Chrysin, Silibinin in combination with ATO generally led to a greater reduction in cell viability than ATO alone, particularly noticeable in GBM43 (34% viability decrease).

## 4. Discussion

The formidable challenge of treating GBM, a highly aggressive and heterogeneous brain tumor, necessitates innovative therapeutic strategies that can overcome its intrinsic and acquired resistance to conventional therapies [2]. Despite showing efficacy in other cancers, ATO monotherapy has had limited success in GBM clinical trials [23]. Phase I/II trials combining ATO with temozolomide and/or radiotherapy showed varied responses that were subtype-dependent [8,18,19,20,21,22,23,24]. Curiously, a study showed synergistic effect of ATO, temozolomide, and the hedgehog signaling pathway inhibitor vismodegib [25]. ATO combination therapy has also been tested in other cancers. For example, the combination of ATO and the EGFR tyrosine kinase inhibitor osimertinib provides a synergistic therapeutic strategy for recurrent and metastatic head and neck squamous cell carcinoma [46], and a clinical trial (NCT03855371) is evaluating a DNA demethylating drug Decitabine and ATO combination to treat acute myeloid leukemia with mutant p53 [47]. These clinical trial results highlighted two primary research directions: identifying biomarkers to predict patient response and developing combination therapies to sensitize resistant tumors [3]. Our study provides compelling preclinical evidence that combining ATO with a MNK1 inhibitor, AUM001, is a promising approach, particularly for overcoming resistance in the aggressive mesenchymal (MES) subtype of GBM [4].

Our results confirmed the strong correlation between ATO sensitivity and the activation of mRNA translation pathways. ATO is known to induce significant cellular stress by generating reactive oxygen species (ROS), causing mitochondrial and endoplasmic reticulum (ER) stress, and inducing DNA damage [48]. Our data suggest that GBM cells with highly active translation initiation pathways are more vulnerable to this ATO-induced stress. In contrast, ATO-resistant models, particularly those of the mesenchymal subtype, exhibit suppressed translation-related pathways and have been shown to overexpress genes associated with translation initiation, including eIF4E [27]. This suggests a mechanism whereby resistant cells, especially MES GSCs, evade ATO’s cytotoxic effects. Therefore, sensitizing these resistant cells requires a targeted disruption of the protein synthesis machinery. The MNK1-eIF4E signaling axis is a critical regulator of cap-dependent mRNA translation, and its dysregulation is a common feature in cancer, promoting the synthesis of oncogenic proteins and therapeutic resistance. These findings suggest that AUM001-mediated inhibition of eIF4E phosphorylation may interfere with survival mechanisms in GSCs treated with ATO. The observed synergy suggests that the MNK1-eIF4E axis could be a factor in the heightened response and reduction in the GSC population when these agents are combined. In our in vitro experiments, the combination of ATO and AUM001 demonstrated a preferential inhibitory effect on the GSC population, even in models that did not exhibit overall synergy in bulk cell population evaluations. We acknowledge that these functional GSC data are currently limited to in vitro assays, and future in vivo validation is necessary to confirm the selective depletion of tumor-initiating cells within the brain microenvironment. Because GSCs are considered key drivers of tumor recurrence and treatment failure [5,6,7], these findings suggest that the combination may target a therapeutically significant sub-population beyond what is reflected in standard bulk viability assays.

Our transcriptomic analysis further revealed that the synergistic interaction between ATO and AUM001 is associated with distinct gene expression signatures. Overall, in synergistic models, we observed an overrepresentation of pathways related to cellular stress responses, which could serve as predictive biomarkers for synergy. An interesting finding from our analysis is the potential role of ion channels in mediating the synergistic effect of the combination therapy. Gene set enrichment and overrepresentation analyses consistently highlighted that genes highly expressed in synergistic models were enriched for terms related to ion transmembrane transporter activity, including amino acid, organic anion, and cation transport. In contrast, models displaying an additive response were dominated by terms related to cell cycle progression, with no significant enrichment for ion channel-related functions. These findings hint that the expression and activity levels of certain transmembrane transporters could play a role in the synergy observed between ATO and AUM001. While the precise mechanisms remain unclear, this association suggests that the relationship between cellular transport and drug synergy in GBM may warrant further exploration. The observed variance in treatment response within established mesenchymal and classical phenotypes underscores the significant intra-subtype heterogeneity inherent in glioblastoma, highlighting the critical need for more refined, functional molecular signatures that transcend broad transcriptional categories to accurately predict therapeutic sensitivity and synergy. We also propose the usage of naturally occurring flavonoids, such as Chrysin and Silibinin, that are themselves non-toxic, but could further sensitize human GBM cells for ATO [49].

Despite these promising observations, several limitations warrant consideration, including the relatively small number of PDX models used and the primarily in vitro nature of the synergy. While recent pharmacodynamic evaluations of AUM001 have demonstrated potent MNK1 inhibition and a favorable half-life in human clinical samples [33], its capacity for effective CNS penetration remains unclear. Further studies are required to establish whether therapeutic concentrations can be achieved in the brain and to assess the potential toxicity of combining these agents. However, the use of ATO at relatively low doses in these combinations may mitigate some safety concerns, as its clinical profile is well-characterized [24], though its interactions with another systemic inhibitor in the context of the GBM microenvironment remain to be fully evaluated.

## 5. Conclusions

In conclusion, our study identifies the combination of ATO and the MNK1 inhibitor AUM001 as a potent, synergistic strategy against GBM, especially for resistant mesenchymal subtypes. The synergy is associated with the inhibition of the MNK1-eIF4E translation initiation axis, which may address a relevant resistance mechanism and correlates with the targeting of the GSC population. We have identified distinct gene expression signatures, including those related to stress response pathways, stemness, and ion channel activity, that are associated with synergy and could be developed into biomarkers for patient selection in future clinical trials. While this study has limitations, such as the number of models tested, it provides a strong rationale for the continued investigation of this combination therapy. Future work will focus on validating these findings in vivo and further exploring the role of GSC-specific vulnerabilities to refine this promising therapeutic approach for GBM patients.

## Data Availability

The original contributions presented in this study are included in the article/Supplementary Materials. Further inquiries can be directed to the corresponding author.

## Supplementary Materials

The following supporting information can be downloaded at https://www.mdpi.com/article/10.3390/brainsci16020121/s1, Figure S1. Drug dose response curves and isobolograms for the ATO and AUM001 combination effects in eight GBM PDX models. Figure S2. Bioinformatics and statistics analysis workflow for investigating ATO response and ATO + AUM001 synergy signatures in GBM PDX cell lines. Figure S3. Correlation between baseline gene expression and synergy scores. Figure S4. Original uncropped Western Blots of Figure 3D. Figure S5. Treatment with Chrysin or Silibinin sensitizes GBM to ATO. Table S1. ATO single agent IC50 and ATO in combination with 6.25 µM AUM001 IC50, Combination Indices and molecular subtypes of 14 GBM PDX models. Table S2. Antibody information.

## References

[1] Gorlia T., van den Bent M.J., Hegi M.E., Mirimanoff R.O., Weller M., Cairncross J.G., Eisenhauer E., Belanger K., Brandes A.A., Allgeier A. (2008). Nomograms for predicting survival of patients with newly diagnosed glioblastoma: Prognostic factor analysis of EORTC and NCIC trial 26981-22981/CE.3. Lancet Oncol..

[2] Dhiman A., Shah Y., Rana D., Garkhal K. (2025). Comprehensive review on glioblastoma: Nanotechnology, immunotherapy and combined therapeutic approaches. RSC Pharm..

[3] Yuan B., Kikuchi H. (2024). Harnessing arsenic derivatives and natural agents for enhanced glioblastoma therapy. Cells.

[4] Bell J.B., Eckerdt F., Dhruv H.D., Finlay D., Peng S., Kim S., Kroczynska B., Beauchamp E.M., Alley K., Clymer J. (2018). Differential response of glioma stem cells to arsenic trioxide therapy is regulated by MNK1 and mRNA translation. Mol. Cancer Res..

[5] Sidhu R.S., Guo S., Wang G., Liu M. (2025). Role of Notch and its oncogenic signaling crosstalk in glioma and glioma stem cells. Gene.

[6] D’Amico M., De Amicis F. (2022). Aberrant Notch signaling in gliomas: A potential landscape of actionable converging targets for combination approach in therapies resistance. Cancer Drug Resist..

[7] Perner F., Berg T., Sasca D., Mersiowsky S.-L., Gadrey J.Y., Thomas J., Kühn M.W.M., Lübbert M. (2026). Therapeutic targeting of chromatin alterations in leukemia and solid tumors. Int. J. Cancer.

[8] Ning S., Knox S.J. (2004). Increased cure rate of glioblastoma using concurrent therapy with radiotherapy and arsenic trioxide. Int. J. Radiat. Oncol. Biol. Phys..

[9] Au W.-Y., Tam S., Fong B.M., Kwong Y.-L. (2008). Determinants of cerebrospinal fluid arsenic concentration in patients with acute promyelocytic leukemia on oral arsenic trioxide therapy. Blood.

[10] Miller W.H., Schipper H.M., Lee J.S., Singer J., Waxman S. (2002). Mechanisms of action of arsenic trioxide. Cancer Res..

[11] Ghaffari S.H., Yousefi M., Dizaji M.Z., Momeny M., Bashash D., Zekri A., Alimoghaddam K., Ghavamzadeh A. (2016). Arsenic trioxide induces apoptosis and incapacitates proliferation and invasive properties of U87MG glioblastoma cells through a possible NF-κB-mediated mechanism. Asian Pac. J. Cancer Prev..

[12] Stevens J.J., Graham B., Dugo E., Berhaneselassie-Sumner B., Ndebele K., Tchounwou P.B. (2017). Arsenic trioxide induces apoptosis via specific signaling pathways in HT-29 colon cancer cells. J. Cancer Sci. Ther..

[13] Haga N., Fujita N., Tsuruo T. (2005). Involvement of mitochondrial aggregation in arsenic trioxide (As2O3)-induced apoptosis in human glioblastoma cells. Cancer Sci..

[14] Zhang J.-Y., Zhang B., Wang M., Wang W., Liao P., Sun G.-B., Sun X.-B. (2017). Calcium homeostasis and endoplasmic reticulum stress are involved in Salvianolic acid B-offered protection against cardiac toxicity of arsenic trioxide. Oncotarget.

[15] Gao X., Liu G., Zhao Z., Tang Y., Hui H., Wang C., Li D., Ma Y., Sun Z., Zhou Y. (2025). Arsenic enhances cervical cancer cell radiosensitivity by suppressing the DNA damage repair pathway. Transl. Cancer Res..

[16] Miller W.H. (2002). Molecular targets of arsenic trioxide in malignant cells. Oncologist.

[17] Fang Y., Zhang Z. (2020). Arsenic trioxide as a novel anti-glioma drug: A review. Cell. Mol. Biol. Lett..

[18] Cohen K.J., Gibbs I.C., Fisher P.G., Hayashi R.J., Macy M.E., Gore L. (2013). A phase I trial of arsenic trioxide chemoradiotherapy for infiltrating astrocytomas of childhood. Neuro. Oncol..

[19] Kumthekar P., Grimm S., Chandler J., Mehta M., Marymont M., Levy R., Muro K., Helenowski I., McCarthy K., Fountas L. (2017). A phase II trial of arsenic trioxide and temozolomide in combination with radiation therapy for patients with malignant gliomas. J. Neurooncol..

[20] Han D., Teng L., Wang X., Zhen Y., Chen X., Yang M., Gao M., Yang G., Han M., Wang L. (2022). Phase I/II trial of local interstitial chemotherapy with arsenic trioxide in patients with newly diagnosed glioma. Front. Neurol..

[21] Grimm S.A., Marymont M., Chandler J.P., Muro K., Newman S.B., Levy R.M., Jovanovic B., McCarthy K., Raizer J.J. (2012). Phase I study of arsenic trioxide and temozolomide in combination with radiation therapy in patients with malignant gliomas. J. Neurooncol..

[22] Kumthekar P., Grimm S.A., Marymont M.H., Mehta M.P., Chandler J., Muro K., Jovanovic B., Helenowski I.B., McCarthy K., Raizer J.J. (2014). Phase II study of arsenic trioxide and temozolomide in combination with radiation therapy in patients with malignant gliomas. J. Clin. Oncol..

[23] Da Silva E.C., Mercier M.-C., Etienne-Selloum N., Dontenwill M., Choulier L. (2021). A systematic review of glioblastoma-targeted therapies in phases II, III, IV clinical trials. Cancers.

[24] Ryu S., Ye X., Olson J.J., Mikkelsen T., Bangiyev L., Lesser G.J., Batchelor T., Nabors B., Desideri S., Walbert T. (2024). Phase I and pharmacodynamic study of arsenic trioxide plus radiotherapy in patients with newly diagnosed glioblastoma. Neurooncol. Adv..

[25] Bureta C., Saitoh Y., Tokumoto H., Sasaki H., Maeda S., Nagano S., Komiya S., Taniguchi N., Setoguchi T. (2019). Synergistic effect of arsenic trioxide, vismodegib and temozolomide on glioblastoma. Oncol. Rep..

[26] Waskiewicz A.J., Flynn A., Proud C.G., Cooper J.A. (1997). Mitogen-activated protein kinases activate the serine/threonine kinases Mnk1 and Mnk2. EMBO J..

[27] Scheper G.C., Proud C.G. (2002). Does phosphorylation of the cap-binding protein eIF4E play a role in translation initiation?: Role of eIF4E phosphorylation. Eur. J. Biochem..

[28] Topisirovic I., Ruiz-Gutierrez M., Borden K.L.B. (2004). Phosphorylation of the eukaryotic translation initiation factor eIF4E contributes to its transformation and mRNA transport activities. Cancer Res..

[29] Furic L., Rong L., Larsson O., Koumakpayi I.H., Yoshida K., Brueschke A., Petroulakis E., Robichaud N., Pollak M., Gaboury L.A. (2010). eIF4E phosphorylation promotes tumorigenesis and is associated with prostate cancer progression. Proc. Natl. Acad. Sci. USA.

[30] Prabhu S.A., Moussa O., Miller W.H., Del Rincón S.V. (2020). The MNK1/2-eIF4E axis as a potential therapeutic target in melanoma. Int. J. Mol. Sci..

[31] Dolniak B., Katsoulidis E., Carayol N., Altman J.K., Redig A.J., Tallman M.S., Ueda T., Watanabe-Fukunaga R., Fukunaga R., Platanias L.C. (2008). Regulation of arsenic trioxide-induced cellular responses by Mnk1 and Mnk2. J. Biol. Chem..

[32] Teneggi V., Novotny-Diermayr V., Lee L.H., Yasin M., Yeo P., Ethirajulu K., Gan S.B.H., Blanchard S.E., Nellore R., Umrani D.N. (2020). First-in-human, healthy volunteers integrated protocol of ETC-206, an oral Mnk 1/2 kinase inhibitor oncology drug. Clin. Transl. Sci..

[33] Gan B.H., Lee L.H., Takeda R., Yasin M., Teneggi V. (2024). Pharmacodynamic evaluation of AUM001/tinodasertib, an oral inhibitor of mitogen-activated protein kinase (MAPK)-interacting protein kinase 1, 2 (MNK1/2) in preclinical models and tissues from a phase 1 clinical study. J. Cancer Sci. Clin. Ther..

[34] Vaubel R.A., Tian S., Remonde D., Schroeder M.A., Mladek A.C., Kitange G.J., Caron A., Kollmeyer T.M., Grove R., Peng S. (2020). Genomic and phenotypic characterization of a broad panel of patient-derived xenografts reflects the diversity of glioblastoma. Clin. Cancer Res..

[35] Zhai K., Mazurakova A., Koklesova L., Kubatka P., Büsselberg D. (2021). Flavonoids synergistically enhance the anti-glioblastoma effects of chemotherapeutic drugs. Biomolecules.

[36] Liu M., Liu X., Qiao J., Cao B. (2024). Silibinin suppresses glioblastoma cell growth, invasion, stemness, and glutamine metabolism by YY1/SLC1A5 pathway. Transl. Neurosci..

[37] Chou T.-C. (2010). Drug combination studies and their synergy quantification using the Chou-Talalay method. Cancer Res..

[38] Hu Y., Smyth G.K. (2009). ELDA: Extreme limiting dilution analysis for comparing depleted and enriched populations in stem cell and other assays. J. Immunol. Methods.

[39] Love M.I., Huber W., Anders S. (2014). Moderated estimation of fold change and dispersion for RNA-seq data with DESeq2. Genome Biol..

[40] Yu G., Wang L.-G., Han Y., He Q.-Y. (2012). clusterProfiler: An R package for comparing biological themes among gene clusters. OMICS.

[41] Wu T., Hu E., Xu S., Chen M., Guo P., Dai Z., Feng T., Zhou L., Tang W., Zhan L. (2021). clusterProfiler 4.0: A universal enrichment tool for interpreting omics data. Innovation.

[42] Liberzon A., Subramanian A., Pinchback R., Thorvaldsdóttir H., Tamayo P., Mesirov J.P. (2011). Molecular signatures database (MSigDB) 3.0. Bioinformatics.

[43] Thomas P.D., Ebert D., Muruganujan A., Mushayahama T., Albou L.-P., Mi H. (2022). PANTHER: Making genome-scale phylogenetics accessible to all. Protein Sci..

[44] Pranali S. (2021). ssGSEA: Gene Signature Enrichment in Individual Samples. https://rpubs.com/pranali018/SSGSEA.

[45] Wickham H. (2009). Ggplot2: Elegant Graphics for Data Analysis.

[46] Hsieh C.-Y., Chang W.-C., Lin C.-C., Chen J.-H., Lin C.-Y., Liu C.-H., Lin C., Hung M.-C. (2022). Combination treatment of arsenic trioxide and osimertinib in recurrent and metastatic head and neck squamous cell carcinoma. Am. J. Cancer Res..

[47] Chen S., Wu J.-L., Liang Y., Tang Y.-G., Song H.-X., Wu L.-L., Xing Y.-F., Yan N., Li Y.-T., Wang Z.-Y. (2025). Arsenic trioxide rescues structural p53 mutations through a cryptic allosteric site. Cancer Cell.

[48] Davison K., Mann K.K., Miller W.H. (2002). Arsenic trioxide: Mechanisms of action. Semin. Hematol..

[49] Gülden M., Appel D., Syska M., Uecker S., Wages F., Seibert H. (2017). Chrysin and silibinin sensitize human glioblastoma cells for arsenic trioxide. Food Chem. Toxicol..

