# Supplementary material for: Arsenic Trioxide and the MNK1 Inhibitor AUM001 Exert Synergistic Anti-Glioblastoma Effects by Modulating Key Translational, Cell Cycle, and Transmembrane Transport Pathways
Source: Brain Sci. 2026 Jan 23;16(2):121. doi: 10.3390/brainsci16020121 (PMC12938313; doi:10.3390/brainsci16020121)
Supplement: Supplementary file 1 [file brainsci-16-00121-s001.zip › brainsci-4077136-supplementary.pdf]

Arsenic trioxide and the MNK1 inhibitor AUM001 exert synergistic anti-glioblastoma effects by modulating key translational, cell cycle, and transmembrane transport pathways

Yue Hao [1†], Charles Shaffer [1,2†], Nanyun Tang [1], Valerie DeLuca [1], Angela Baker [1], Michael E. Berens [1*]

[1] Clinical Genomics and Therapeutics Division, Translational Genomics Research Institute, Phoenix AZ 85004

[2] Beckman Research Institute, City of Hope, Duarte, CA 91010

† These authors contributed equally to this work

* Correspondence: mberens@tgen.org

Supplementary Figures and Tables

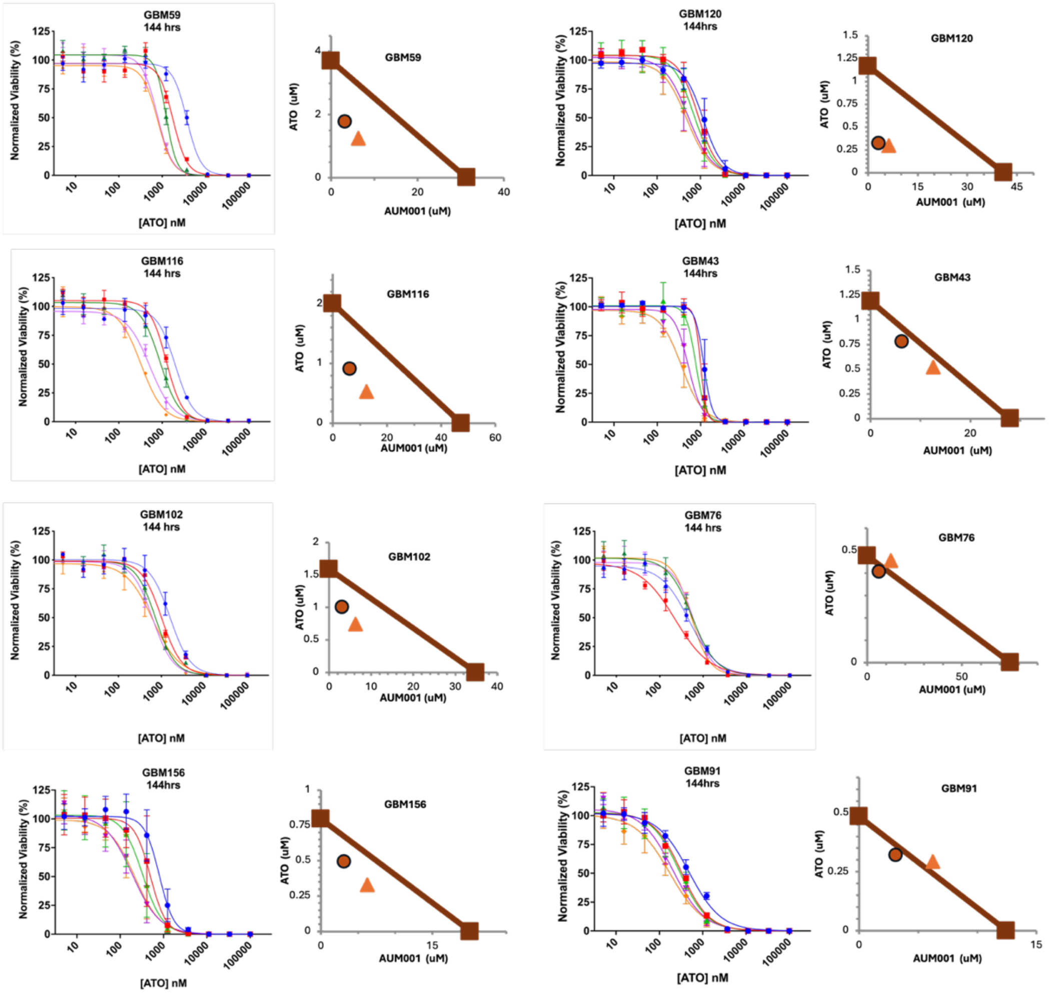

Figure S1. Drug dose response curves and isobolograms for the ATO and AUM001 combination effects in eight GBM PDX models. See also Figure 2.

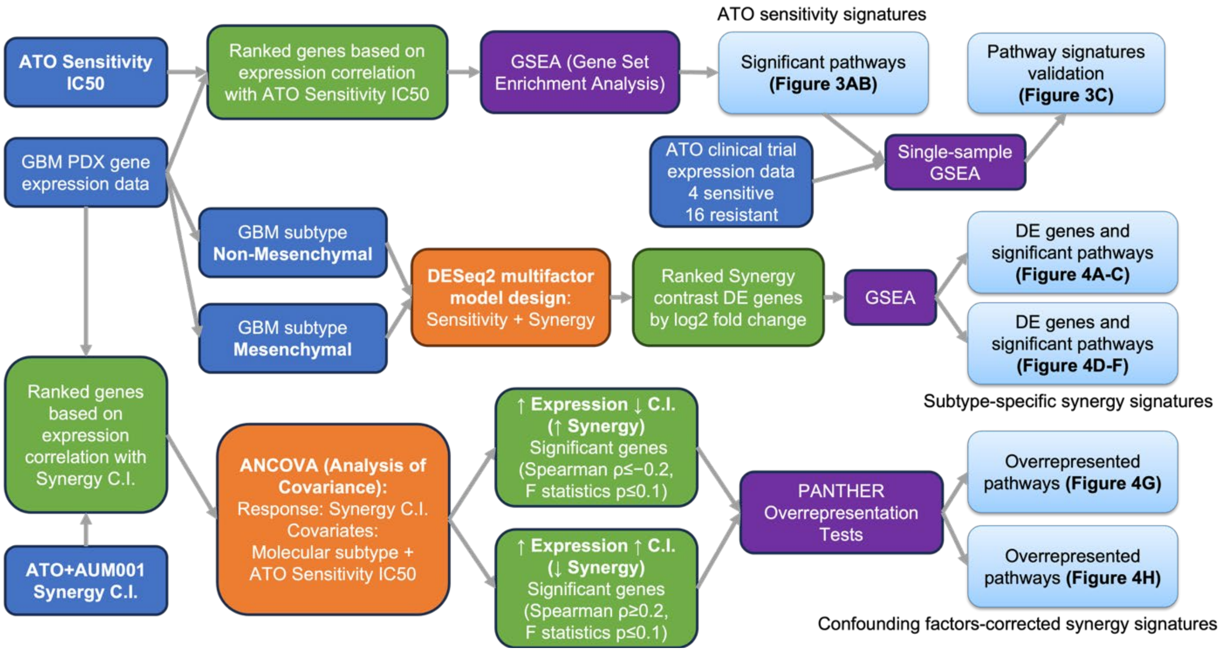

Figure S2. Bioinformatics and statistics analysis workflow for investigating ATO response and ATO+AUM001 synergy signatures in GBM PDX cell lines.

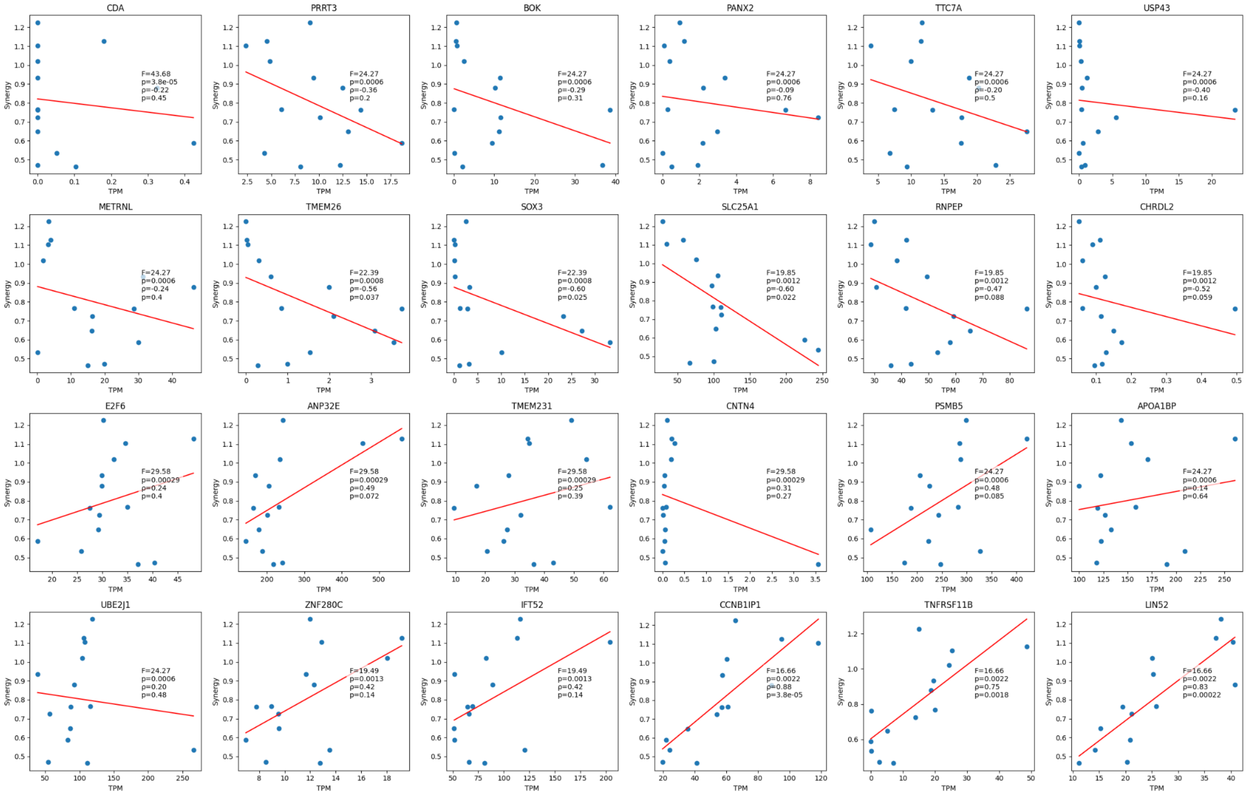

Figure S3. Correlation between baseline gene expression and synergy scores. Scatter plots depicting the relationship between baseline gene expression (TPM) and synergy scores for 24 individual genes. Each point represents an individual sample. The red line indicates the linear regression fit. The F-statistic and p-value for each correlation are displayed on the respective plots.

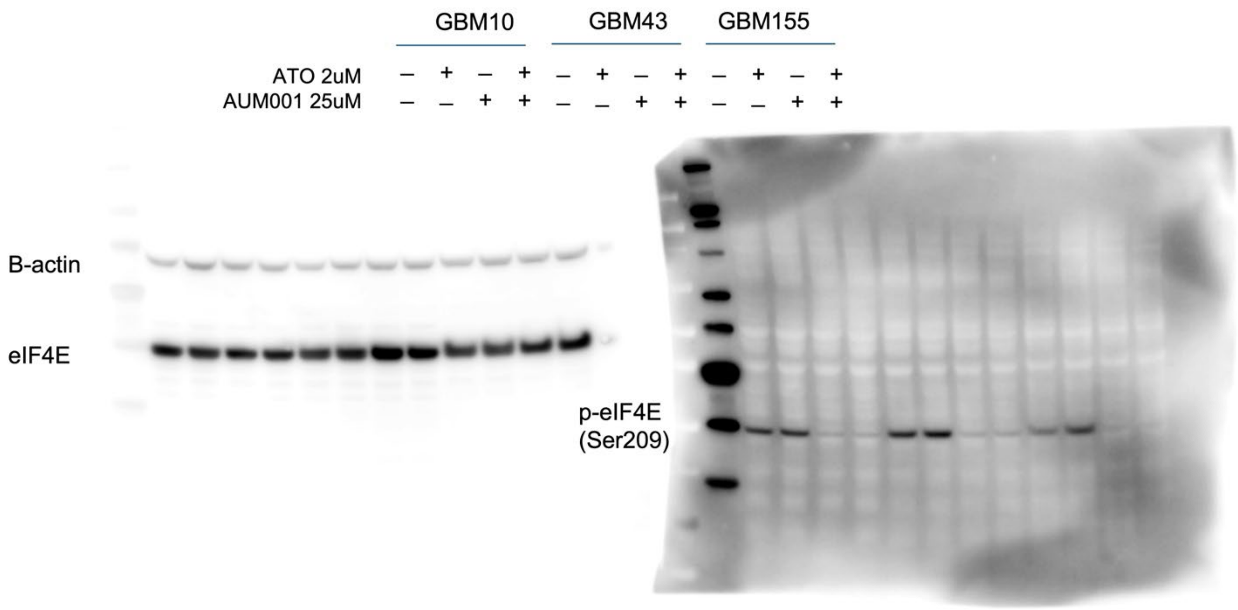

Figure S4. Original uncropped Western Blots of Figure 3D.

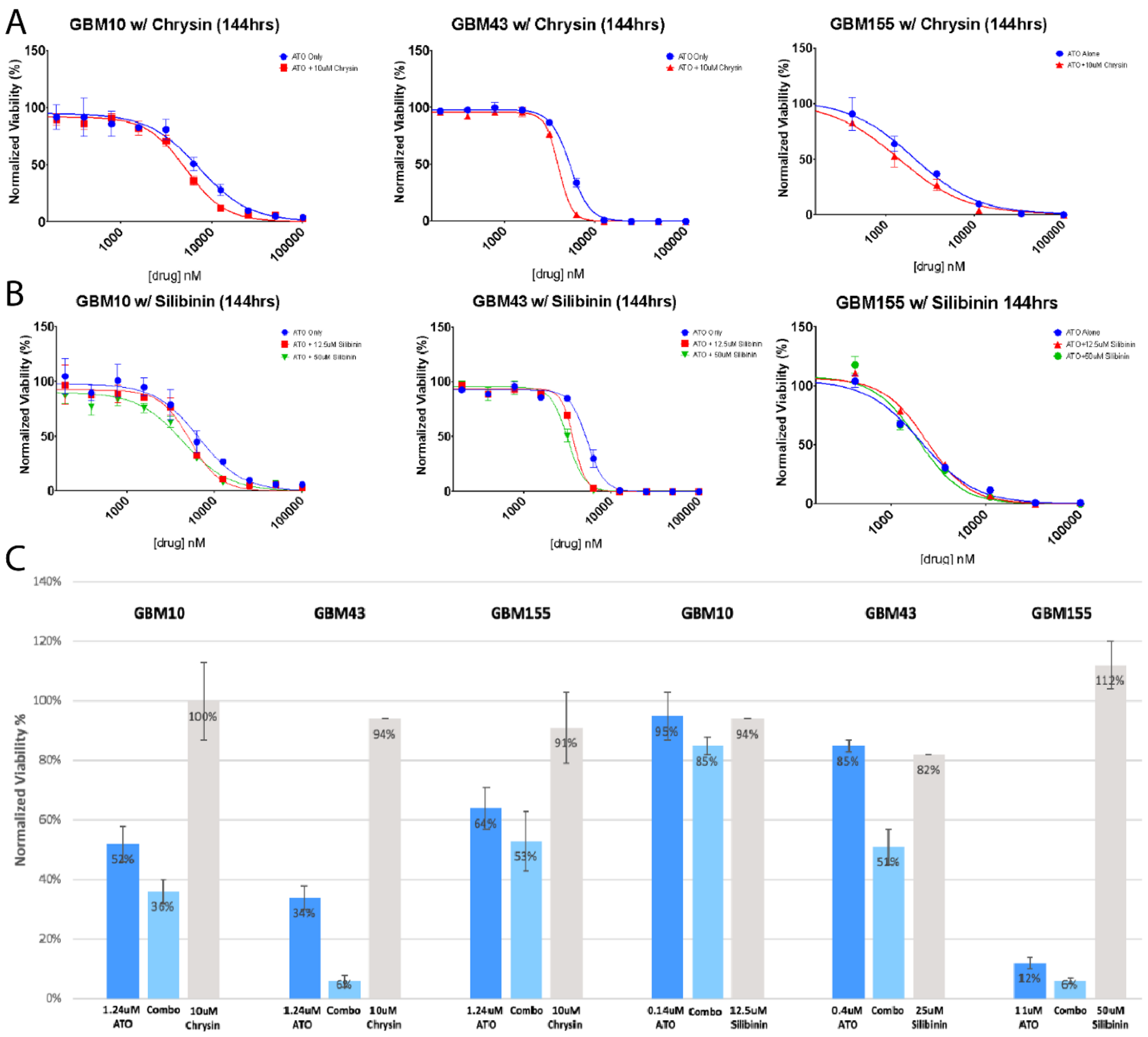

Figure S5. Treatment with Chrysin or Silibinin sensitizes GBM to ATO. GBM10, 43, and 155 were dosed with ATO concentrations ranging from 100 μmol/L to 0.565 nmol/L either alone, in combination with Chrysin (A), or Silibinin (B). The cell viability of the singular treatments and combinations were recorded to determine the degree of the combination effects (C). Combination treatments involving Chrysin reduced cell viability by 9-28% compared to ATO alone, treatments involving Silibinin similarly reduced viability by 6-34%. Error bars show standard deviation calculated from twelve replicates per condition (three biological replicates each with four technical repeats).

Table S1. ATO single agent IC50 and ATO in combination with 6.25 µM AUM001 IC50, Combination Indices and molecular subtypes of 14 GBM PDX models.

Sample ID	ATO IC50	ATO IC50 w AUM001	C.I.	Synergy	Level*	Molecular_subtype*
X76	0.48	0.405	0.934	Synergistic	Mild	Classical
X91	0.486	0.193	1.104	Additive	Nonsyn	Classical
X156	0.797	0.33	0.724	Synergistic	Strong	Classical
X155	0.915	0.892	1.226	Additive	Nonsyn	Classical
X120	1.166	0.712	0.763	Synergistic	Mild	Proneural
X43	1.186	0.777	0.879	Synergistic	Mild	Mesenchymal
X38	1.25	0.665	1.02	Additive	Nonsyn	Classical
X102	1.59	0.745	0.648	Synergistic	Strong	Classical
X10	1.707	1.267	1.127	Additive	Nonsyn	Mesenchymal
X116	2	0.911	0.587	Synergistic	Strong	Mesenchymal
X6	2.228	0.606	0.766	Synergistic	Mild	Classical
X126	3.53	1.32	0.471	Synergistic	Strong	Classical
X59	3.7	1.239	0.534	Synergistic	Strong	Mesenchymal
X108	5.36	1.745	0.464	Synergistic	Strong	Mesenchymal

* Synergistic level: C.I. > 1, non synergistic; 0.75 < C.I. < 1, mild synergy; C.I. < 0.75, strong synergy.
* GBM molecular subtypes are from (Vaubel et al. 2020).

Table S2. Antibody information.

Antibodies	Source	Identifier	Dilution for WB
MNK1	Cell Signaling	2195	1:1000
B-Actin	Cell Signaling	4970	1:20000
